# Emergency Verbal Consent for Intrapartum Research: A Grounded Theory Study

**DOI:** 10.1111/1471-0528.17997

**Published:** 2024-11-07

**Authors:** Carol Bedwell, Wendy Taylor, Caroline Cunningham, Andrew D. Weeks, Dame Tina Lavender

**Affiliations:** ^1^ Centre for Childbirth, Women's and Newborn Health, International Public Health Liverpool School of Tropical Medicine Liverpool UK; ^2^ Focussed Care West Gorton Manchester UK; ^3^ Liverpool Women's Hospital Liverpool UK; ^4^ Department of Women's and Children's Health University of Liverpool Liverpool UK

**Keywords:** assent, consent, emergency, obstetric, research, verbal

## Abstract

**Objective:**

To understand the experiences of women, birth partners and health professionals of verbal followed by retrospective written consent in a prospective cohort study of a device to manage postpartum haemorrhage (PPH).

**Design:**

Grounded Theory.

**Setting:**

Tertiary facility in North‐West England, UK.

**Sample:**

We used purposive and theoretical sampling to recruit 51 participants; 12 women, 12 birth partners, 16 obstetricians and 11 midwives.

**Methods:**

Semi‐structured interviews were conducted, using a topic guide for focus, until data saturation was achieved. Data were analysed using framework analysis technique.

**Results:**

Most women wanted sufficient information to make a decision at the time of the event, rather than in advance, and preferred not to be overwhelmed with detail. A key factor in making the decision to participate was a positive and trusting relationship with the attending obstetrician. Obtaining consent for research in emergencies was viewed by obstetricians as requiring a different approach and more challenging than consent for standard procedures in an emergency.

**Conclusions:**

This is one of the first studies to explore verbal followed by retrospective written consent processes with women, clinicians and observers. This was acceptable to all, however information needs to be appropriate, and those discussing consent require adequate training (199/200).

## 1 Introduction

Informed consent is a key principle in research participation [1]. Ethical procedures require that potential participants receive adequate information and have time to consider participation prior to giving written consent. In emergency situations, this may not be possible and has the potential to delay treatment. In order to improve outcomes for women/patients, it is vital that research into treatments and management of emergency situations is completed. Labour and childbirth is a particular example of this, as obstetric emergencies may occur rapidly requiring immediate intervention. Due to the emergency situation, labour pain and disorientation due to medication women may be classed as vulnerable [2, 3]. Additionally, some may be unable to provide consent due to incapacity, for example if there is loss of consciousness. In situations where women are unable to give consent due to incapacity, the Health Research Authority (HRA) allows for enrolment without prior consent, subject to certain conditions [4]. Other studies have used proxy consent procedures, with consent given by a representative followed by written consent [5]. Where there is no incapacity, current guidance suggests that verbal consent may be obtained in acute situations, with informed written consent obtained in retrospect [3]. This approach was taken in a Phase 2 clinical trial in which a novel device known as the PPH Butterfly was used to treat postpartum haemorrhage (PPH) (PPH Butterfly Phase II study, II‐LA‐0715‐200 008) [6].

PPH is a common obstetric emergency following birth and the most common cause of maternal death worldwide. The main cause is uterine atony which can be managed by use of uterotonic drugs, bimanual compression and surgery. Although highly effective, bimanual compression has limited use because it is invasive, uncomfortable for the woman and can be difficult to maintain. The PPH Butterfly is a new device designed to facilitate bimanual compression, making it less invasive [7]. It is introduced vaginally and consists of a platform which sits against the cervix and allows for abdominal pressure to compress the uterus, thereby reducing bleeding.

Alongside the main study, a pre‐planned qualitative exploration of experiences of the use of the PPH Butterfly was undertaken. This enabled greater understanding and multiple perspectives to be obtained. The aim of this study was to understand the experiences of women, partners and health professionals around the issue of taking verbal consent, followed by retrospective written consent, for the main study.

## 2 Methods

**FIGURE 1:**
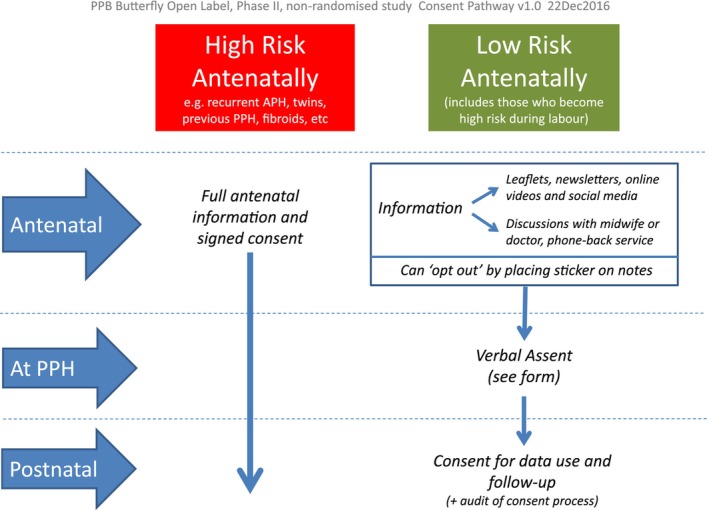
Consent pathways for the PPH Butterfly Study.

All women attending Liverpool Women's Hospital for antenatal care between January and December 2018 received information about the study in the form of a participant information sheet. These were distributed in antenatal clinics in the community and hospital. Posters and leaflets informing women about the study were also displayed in antenatal clinics. Contact numbers for researchers and opportunities to discuss the study with them were given to all women. Women at known high risk of PPH, such as women with twin pregnancies, were approached for written informed consent by a researcher in the antenatal period. If a woman indicated that she did not want to be approached further, a sticker was placed in her notes to alert clinicians to this. See Figure 1 for the recruitment process.

**FIGURE 2:**
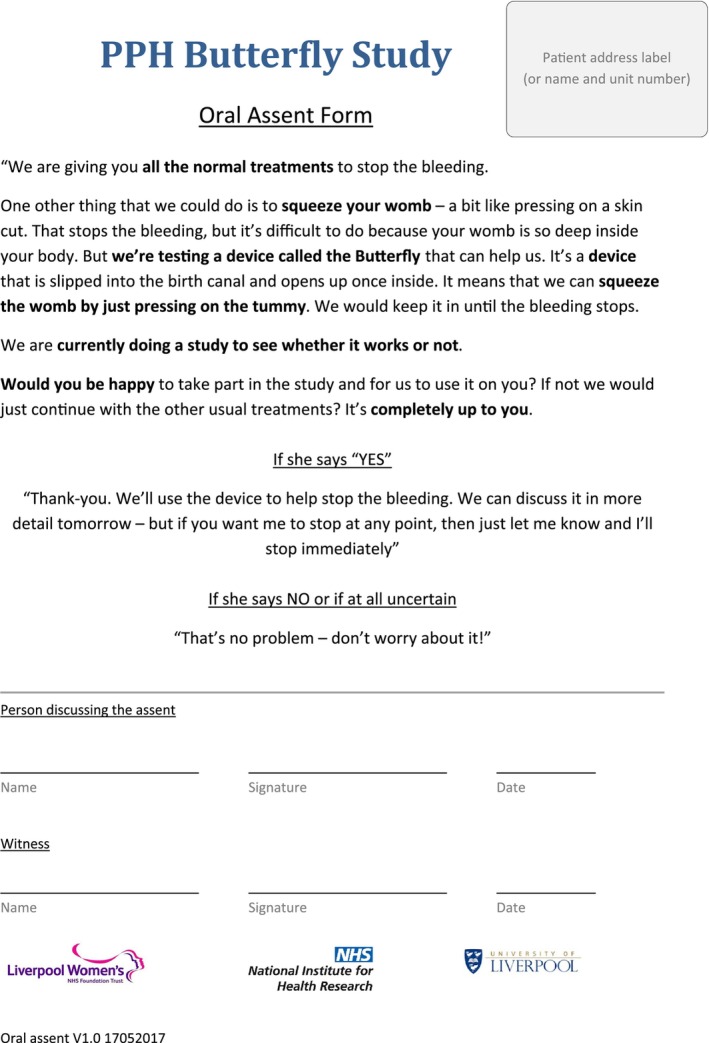
Form used by recruiting clinicians for emergency intrapartum verbal consent.

Obstetricians trained in use of the PPH Butterfly device were also trained in gaining consent for research in an emergency situation. This was provided as part of the overall intervention training process by a researcher (WT). In the event that a woman experienced a PPH, the attending clinical team would administer first aid to her, gaining intravenous access, taking blood for grouping and administering oxytocin. During these initial procedures, the recruiting obstetrician would also discuss the use of the device and gain verbal consent to use the PPH Butterfly device should the bleeding continue. A script was provided to the recruiting physicians and was signed by them after recruitment (see Figure 2). The obstetricians were instructed to only use the device if the woman was certain that she wanted to continue; if she declined, appeared hesitant, uncertain or confused, then normal care would continue without use of the device. The time taken to discuss consent was typically 1–2 min. In those whose bleeding continued despite the initial measures and who provided verbal consent, the device was inserted, and uterine compression commenced alongside normal care. The clinician was instructed to remove the device if at any point the participant requested.

Following management of the situation and after sufficient recovery time, the woman was approached by a trained research midwife to discuss the study and obtain written consent. Women giving consent to the study were then asked to complete a questionnaire, which included a Likert scale question on the acceptability of the consent process. Data were cleaned and entered into SPSS; analysis provided descriptive statistics of the responses to this question.

The qualitative aspect of the study explored the consent process through the views of the women participants, their birth partners, midwives and obstetricians.

The qualitative approach was underpinned by Straussian grounded theory methodology [8], allowing for the simultaneous collection and analysis of data. This approach was appropriate given the social interactions involved in the consent processes, and the need for acknowledgment of the a priori experiences of the researchers. This theory is ‘grounded’ in observation and utilises questions that are not confining or static. As data are gathered, core theoretical concepts become identifiable and begin to develop [9]. The grounded theory approach provided flexibility in that the number of participants interviewed was open to change as new findings were discovered, and/or when data saturation was achieved, whilst ongoing data collection introduced new information thereby changing the direction of interviews. By immersing themselves in the data, the researchers were able to explore what was important to the participants.

Inclusion criteria were that the participant was present at the birth and during the use of the PPH Butterfly device. Women included in the main study were provided with a participant information sheet by the midwife obtaining retrospective consent. If women were agreeable, a different research midwife (WT) contacted them by their preferred method, after a time of at least 24 h had elapsed. Interviews were completed at a time of the woman's choosing, usually at her home address, following informed written consent. If a woman gave consent to interview, then a further participant information sheet was provided to their birth partner and a similar process followed. Obstetricians and midwives present when the device was used were provided with participant information sheets. If they gave consent, face‐to‐face interviews were conducted within a private environment in the hospital setting. The sample size for all groups was guided by data saturation [7]; that is when no new themes emerged from the data.

Interviews with all groups were semi‐structured, with a separate topic guide for each group by the research team, enabling focus to be maintained during the interview. Data collection was completed in the 3 weeks following birth, in order to minimise recall bias.

Interviews were digitally audio recorded, transcribed, and the data analysed using a framework analysis approach [10], which enabled open, axial and selective coding [8] to be conducted in a systematic and transparent way. This approach is useful for facilitating the constant comparative techniques required in grounded theory [11]. The data from each group of participants were analysed separately and then merged at the axial stage to finalise the themes. Data collection ceased when data saturation was achieved as agreed by the two researchers completing analysis (WT, CB). At this point, data were becoming repetitive, with no new themes emerging. Data were collected between January and November 2018.

### 2.1 Patient and Public Involvement

In line with INVOLVE guidance, a Public Engagement Panel (PEP) participated in the development of the original device and continued to provide advice throughout the clinical research phase. The co‐ordinator of this group was a full member of the Trial Management Group that met each month and provided ongoing liaison with the PEP. The PEP provided initial input into the consent and recruitment process, met the principal investigator and research midwives 6‐monthly during the study to provide ongoing advice and feedback, and again at the end to discuss conclusions and overall assessment of the outcomes. A public meeting attended by hospital staff, recruiters, researchers and patient participants was held in January 2020 to discuss the results and provide input into the final conclusions.

### 2.2 Reflexivity

All of the research team have previously undertaken clinical training in midwifery or medicine and have gained consent for clinical trials; their interpretations may differ from others. Four of the authors are female; three have given birth. The interviewing midwife (WT) has previous personal experience of NHS maternity care, and this may have helped build an honest and open relationship with those being interviewed. Memos were kept throughout data collection and analysis to enable a clear audit trail. The PEP provided non‐institutional insights into the research protocols and results.

## 3 Results

A total of 57 women participated in the main study (PPH Butterfly II). The clinical results have been published elsewhere [6]. Six women were approached at the time of the PPH but declined to give verbal consent for the device use with reasons recorded as ‘not interested’ (*n* = 4), ‘did not like idea of new treatment’ (*n* = 1) and ‘insufficient analgesia’ (*n* = 1). Of the remaining 57 women who gave verbal consent at the time of the PPH, all provided postnatal written consent. In the follow‐up questionnaire, 89% (*n* = 50) either ‘agreed’ or ‘completely agreed’ that they were happy with the way in which they were recruited to the study (see Table 1). Only 4% (*n* = 2) of women were not satisfied with the consent process.

**TABLE 1:** Likert scale of acceptability of consent process.

Response	I was happy with the way that I was recruited to this study
Completely disagree	0 (0%)
Disagree	2 (4%)
Neither agree nor disagree	4 (7%)
Agree	33 (59%)
Completely agree	17 (30%)
Unobtainable	1

The qualitative study included a total of 51 participants; 12 women with whom the device was used, 12 of their birth partners, 16 recruiting obstetricians and 11 attending midwives. Of the 12 women included in the qualitative study, 50% (*n* = 6) had an epidural in situ at the time of the PPH whilst the other 50% (*n* = 6) had received opiate or inhalational analgesia. The majority gave birth in lithotomy position with more than half being operative births (i.e., ventouse or forceps), performed on delivery suite. All obstetricians interviewed were ST3 or above and several had used the device on more than one occasion. The majority had vast experience of dealing with PPH and had used bimanual compression frequently in the past. Interviews lasted between 7 min to 1 h in length. Participants' responses relating to both the PPH and the use of the device were published with the clinical study results [6], but responses related to the consent process are presented here separately.

The four key themes identified were the balance of information, trusting relationships, making the decision and a different situation.

### 3.1 Balance of Information

Participants in all groups interviewed believed the consent process was acceptable, and the information provided was sufficient given the circumstances.

‘*Considering I was bleeding, they discussed it very calmly, they went through what it was that was being used, and I had absolutely no objections because it's it was explained that it was something to help the stop the bleeding and I was bleeding, so I was like ‘yes, that's fine*.’ (*Woman 04*)

The approach was viewed as reasonable and neither women nor their birth partners felt that there was any pressure to participate.

‘*It didn't seem like there was any pressure. It was sort of explained that it was a new device erm that was being trialled that was to help people and erm it could be beneficial*.’ (*Birth partner 5*)

Women suggested that they needed only to be provided with sufficient relevant pertinent information to make a decision.

‘*I think more than enough information at the time. I think if I'd been given too much information it would have bogged me down. You don't need to know all this information now, you need to know enough information to give informed consent*.’ (*Woman 02*)

Despite provision of information in advance, many women were unaware of the study at the time of labour. However, women stated they did not necessarily want information about something that may not happen to them. This was even in the case for one woman who had previously experienced a PPH.

*Probably not [want information] because then you're receiving information about something that may or may not be used, and I'd rather be told if it's being used in the situation where it needs to be used, as opposed to just having the information* ‘*oh this is something that could be used*.’ (*Woman 04*)

Partners conversely would have rather received information in advance as they were mostly unaware of the risks of PPH.

‘*I think shouldn't this information have been given to her before she er went into labour so even if it was in her notes, you know, this could be an option, do you consent that if it needs be in an emergency that we use this*?’ (*Partner 10*)

Only one woman had provided consent in advance, at antenatal clinic, and was keen for the device to be used.

### 3.2 Relationship

For both women and birth partners, the relationship with the obstetrician caring for the woman was pivotal in the decision to provide verbal consent. All believed the device would only be suggested for use if it were in the woman's best interests and there was considerable trust evident between the woman, her partner and the obstetrician.

‘*We quickly erm had a lot of faith in the professor, so when it was suggested, yeah I was concerned but the moment that he was willing to do it, I…I…I knew that she wouldn't be in any danger*.’ (*Birth partner 1*)

Midwives and obstetricians also believed the relationship was important in taking consent in this situation. One obstetrician described the differences between knowing the women and being a stranger entering the room to take consent.

‘*I've been in that situation with attempting to recruit for this study already where I have come in, the lady has still been bleeding after oxytocin, and you are a stranger coming into the room, erm explaining about a study that she's never heard of, and she's afraid because she's been told that she's still bleeding despite the standard treatment, and erm understandably, the woman's said ‘no’ in that erm situation*.’ (*Obstetrician 5*)

### 3.3 Making the Decision

Women viewed the decision to take part as straightforward and none of them recall consulting their birth partner in making the decision.

‘*I thought the doctors and midwives and then the lady who explained it was really like calm, she was good, explained it all. I can't really remember what she said now but I remember thinking at the time, yeah that sounds good, we'll go for this*.’ (*Woman 05*)

The advantages of understanding the various viewpoints around decision‐making were evident in women's and partners' recall of events. Some birth partners suggested that their partner was not fully aware given that she was in labour and had received analgesia.

‘*She was obviously on gas and air and she'd obviously had injections or whatever, so really she might not have been of the right mind to make a conscious choice like that*.’ (*Birth partner 10*)

However, his partner felt she had sufficient understanding to make a decision.

‘*I might have been a bit out of it with the drugs, but I was aware of what was going on and erm I just I had my eyes closed like a lot of the time. I just remember listening to what people would say were saying to me*.’ (*Woman 10*)

One midwife was concerned that a woman she was caring for would not necessarily understand the information.

‘*She was bilingual, so we were a little bit concerned about that, but she definitely understood ‘cos she asked questions back. I was concerned at him [obstetrician] consenting her for that whether she understood completely that he was gonna put this device inside her. I was surprised that she consented, but she did consent. Quite, quite clearly*.’ (*Midwife 8*)

All felt that women were provided with sufficient information and that women freely made the decision to participate. Women, partners and obstetricians had a clearer recall of events as the midwives present were busy monitoring the woman, note taking and preparing drugs.

### 3.4 A Different Situation

Although they were used to taking consent for treatments in emergency situations, many obstetricians felt that gaining consent for research in an emergency differed, requiring a higher level of description.

‘*So, what I'm saying is a lot of verbal consent in emergencies is described in terms that might be vague, because it's an emergency, but also to not cause more psychological distress to the woman. Therefore, with consent for the PPH Butterfly, we allow people better to understand what is happening*.’ (*Obstetrician 11*)

The training in taking consent in these situations was viewed as important by the obstetricians.

‘*But more than anything it's [training] helped to take consent for research and the wording of how to actually say it. More than anything this was important*.’ (*Obstetrician 14*)

## 4 Discussion

This research involved women who had capacity to consent but were unable to go through the full consent processes due to the emergency nature of the situation. Whilst there is some debate around competence to consent in labour, women in this study appeared to understand the information given during the emergency situation. They also found the initial verbal consent process acceptable in the circumstances, and many women felt the decision to participate was straightforward. It was interesting that some women made a conscious choice that they did not want information in advance of an emergency occurring, and some had chosen not to engage with information they had been provided with antenatally. For clinicians who were seeking consent from the women, this meant that many women did not have a prior depth of understanding of the study. The clinician obtaining consent therefore had to convey the main study details within a short timeframe, which some found challenging.

### 4.1 Strengths and Limitations

A strength of this study was the 360° view of the consent process that not only included those involved in the discussion, but also those observing. Triangulation of the data allows for greater confidence in the findings [12]. The inclusion of birth partners views extends previous studies findings related to women and health professionals [5, 13]. Data were collected within 3 weeks of the event, thus avoiding recall bias.

The main study limitation was that only the views of women who gave consent to take part were obtained and represented here. We do not know the views of the women who declined to participate, and whether they were satisfied or even traumatised by the consent process. This selection bias could have distorted the results as well as missing significant harm. Future studies would benefit from asking these women's opinions as well. Furthermore, this study only provides data on this specific study conducted in this specific culture. The process may not therefore be generalisable to other emergency research studies or cultures where expectations and relationships between clinicians and woman are different. A careful prior analysis of the situation along with the involvement of a local PEP is critical in creating an appropriate consent pathway for each study.

### 4.2 Interpretation

This study followed RCOG guidance for obtaining consent for research in acute situations [3]. The process of obtaining verbal consent with subsequent written consent was found to be acceptable to women and birth partners. This reflects the information‐giving process for taking verbal consent for clinical procedures, despite the fact that many obstetricians perceived that more detail was required for research. Women in this study, however, believed they had been provided with adequate information with which to make a decision, even though there was not perfect recall of the exact information given. This differs from other studies where some women did not recall their involvement in research [5]. This may be due in part to the fact that half the women in this study chose epidural for pain relief and therefore may not have experienced disorientation due to systemic analgesia. However, even women who chose opioid methods of analgesia were able to recall giving consent, if not the detail. The balance of information given was important; women did not want to be overwhelmed with information, rather they preferred to receive the key points on which to base their decision, reflecting the findings of others [14, 15]. In practice, information requirements are likely to vary between women, dependent on their individualised needs and situation at the time [2].

Our findings agree with a previous smaller study of women who gave verbal assent for another emergency intrapartum intervention (delayed cord clamping at preterm birth) that also found that the limited information provided was sufficient to make a decision regarding participation [16].

The information needs of women appeared to be dependent on the evolving situation, with women in this study mostly not wanting prior information about the study. Whilst it is recognised that women do seek out information about pregnancy and birth [17], this may relate to normal process, rather than complications of birth or research. Women may be employing this as a strategy to preserve their plans for normality in labour and birth, with consideration of alternatives only when necessary. A systematic qualitative review by Downe [18] suggests that whilst women do not want interventions, they do want a safe birth; this may explain how and when women choose to interact with information and choices for care. Birth partners felt that women should have information in advance; however, they were less aware of potential complications of labour and birth. Dynamic consent, an electronic resource for providing information and recording continuously updated birth plans, has been suggested as a way of capturing this process [19].

In determining study participation, the relationship and degree of trust between the woman and the obstetrician seeking consent was paramount, reflecting previous findings [5, 15, 20, 21]. In this study, obstetricians also agreed the relationship was important and felt more comfortable discussing the study with a woman they had built a relationship with, rather than someone they had just met.

Communication was an important element in the consent process, and the verbal nature of the information delivery may have enabled the key points of the intervention to be highlighted in a concise and understandable way. However, this is dependent on the skills and equipoise of the clinician to ensure balanced information is given. Self‐perceived clinical competence, team support and time factors are all elements which can affect information giving and clinical decision‐making in emergencies [22]. Clinicians clearly felt less comfortable in gaining consent for research than for clinical care, despite being experienced in obtaining consent for emergency treatments. The additional training given in taking consent for research in these situations was welcomed by clinicians and may have increased study participation. The wording provided on the consent form (Figure 2), developed in conjunction with patient representatives, was especially valued as providing a simple but appropriate guide for the emergency situation. The required witness signature provided validity, an important aspect for all parties. This is an area to be considered for successful recruitment in studies using this approach to consent.

In an emergency intrapartum situation, time is critical, and it would be unacceptable to delay emergency treatment in order to discuss consent for research. Women in the COPE study, an ongoing randomised trial comparing two first line PPH medications, do not consider verbal consent to be approriate [4]. For that situation, alternative consent processes are needed. For example, in the COPE study, no consent is taken but postnatal consent for use of data and follow‐up are requested the following day. In the first 161 recruits to that study only 4% were dissatisfied with this process of consent [4]. By way of contrast, in this study the PPH Butterfly device was used as a second line therapy for those who continued to bleed despite initial oxytocin treatment. This gave a brief window of opportunity to discuss consent for the research. Despite being only a very short time period, it is similar to the time taken for use of standard therapeutic non‐research interventions and the women in this study felt that it was adequate for them to give a response. The sensitivity and communication skills of the recruiting clinicians were critical in this situation, as was their training to exclude any woman who showed uncertainty about participating.

Although the COPE [4] and Butterfly studies may seem similar, there were subtle differences both in the familiarity of the intervention and the time available for discussing consent which made it appropriate to have two different consent methods. This underlines the need for careful customisation of the emergency consent process with patient representatives during study preparation. Each study needs to consider the safety/novelty of the intervention, whether the intervention replaces or is in addition to normal care, and the time available for discussion in determining the appropriate process. For example, if the intervention in this study had been higher risk, replaced normal care (rather than being additive) or been first line, then prior consent of all women so as to be ready for the small number of women requiring the intervention might have been more appropriate. Future guidelines should focus on these nuances rather than providing a one‐size‐fits‐all solution.

## 5 Conclusion

This study is unique in exploring the views of women participants, the obstetricians taking consent and midwives and birth partners observing the consent process. The verbal consent with retrospective written consent process was acceptable to women, birth partners and health professionals in this study, and is a suitable approach for recruitment in similar contexts (4219).

## Author Contributions

This study was designed by C.B., A.D.W. and T.L. as part of the PPH Butterfly phase 2 study, led by A.D.W., W.T. conducted the staff training; interviews and data analysis were carried out by W.T., C.C. and C.B. and supervised by T.L. C.B. wrote the first draft of the manuscript; W.T., C.C., A.D.W. and T.L. provided comments and approved the final manuscript.

## Disclosure

A.D.W. is one of the inventors of the PPH Butterfly. The patent is held by his employer, the University of Liverpool, but he would receive a share of royalties on any future sales.

## Ethics Statement

The clinical study was approved by the Health Research Authority (HRA) and the North West Liverpool Central Research Ethics Committee (Ref [17]/NW/0373). The qualitative study was approved by the Office for Research Ethics Committees Northern Ireland (17/NI/0140). Both studies were sponsored by the University of Liverpool.

## Conflicts of Interest

The authors declare no conflicts of interest.

## Data Availability

The data that support the findings of this study are available from the corresponding author upon reasonable request.
